# A multireflection and multiwavelength residual stress determination method using energy dispersive diffraction

**DOI:** 10.1107/S1600576718004193

**Published:** 2018-05-09

**Authors:** Marianna Marciszko, Andrzej Baczmański, Manuela Klaus, Christoph Genzel, Adrian Oponowicz, Sebastian Wroński, Mirosław Wróbel, Chedly Braham, Habib Sidhom, Roman Wawszczak

**Affiliations:** a AGH University of Science and Technology, ACMIN, Aleja Mickiewicza 30, 30-059 Kraków, Poland; b AGH University of Science and Technology, WFiIS, Aleja Mickiewicza 30, 30-059 Kraków, Poland; c Abteilung für Mikrostruktur- und Eigenspannungsanalyse, Helmholtz-Zentrum Berlin für Materialien und Energie, Albert-Einstein-Strasse 15, 12489 Berlin, Germany; d AGH University of Science and Technology, WIMiIP, Aleja Mickiewicza 30, 30-059 Kraków, Poland; e Arts et Métiers ParisTech (ENSAM), PIMM, 151 Boulevard de l’Hôpital, 75013 Paris, France; f Université de Tunis, Laboratoire de Mécanique, Matériaux et Procédés, LAB-STI-03 ESSTT, 5 Avenue Taha Husseïn BP 56, Bab Menara, 1008 Tunis, Tunisia

**Keywords:** residual stress, synchrotron radiation, hexagonal structures, energy dispersive diffraction, multireflection grazing incidence diffraction, MGIXD, multireflection and multiwavelength X-ray diffraction, MMXD

## Abstract

The main focus of the presented work was the investigation of structure and residual stress gradients in the near-surface region of materials studied by X-ray diffraction. The multireflection method was used to measure depth-dependent stress variation in near-surface layers of a Ti sample (grade 2) subjected to different mechanical treatments. First, the multireflection grazing incidence diffraction method was applied on a classical diffractometer with Cu *K*α radiation. The applicability of the method was then extended by using a white synchrotron beam during an energy dispersive (ED) diffraction experiment. An advantage of this method was the possibility of using not only more than one reflection but also different wavelengths of radiation. This approach was successfully applied to analysis of data obtained in the ED experiment. There was good agreement between the measurements performed using synchrotron radiation and those with Cu *K*α radiation on the classical diffractometer. A great advantage of high-energy synchrotron radiation was the possibility to measure stresses as well as the *a*_0_ parameter and *c*_0_/*a*_0_ ratio for much larger depths in comparison with laboratory X-rays.

## Abstract 2

Multireflection grazing-incidence X-ray diffraction was used to investigate the structure and residual stress gradients in the near-surface region of mechanically treated titanium samples. The development of this method by using a white synchrotron beam during an energy dispersive diffraction experiment is proposed.

## 1. Introduction   

Residual stresses together with microstructure are one of the most important parameters for materials characterization. The stress state and material properties are usually heterogeneous in the near-surface volume of machined samples. This is why the design and determination of these properties by appropriate experimental methods is of great importance. X-ray diffraction stress analysis (XSA) in reflection mode is a non-destructive technique that is commonly used because of its many advantages (Noyan & Cohen, 1987 ▸; Hauk, 1997 ▸; Reimers *et al.*, 2008 ▸). Especially important and useful are the XSA methods that allow for residual stress determination in well defined layers under the surface or within the sample volume.

**Figure 1:**
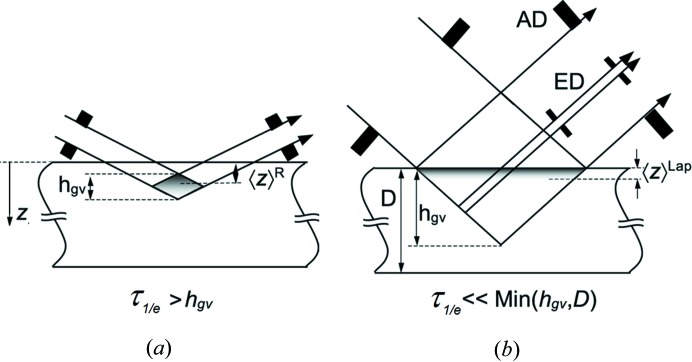
Height of the gauge volume (*h*_gv_) immersed in the sample compared with the information depth in real space 〈*z*〉^R^ and in Laplace space 〈*z*〉^Lap^. Different modes of X-ray and synchrotron diffraction are shown: (*a*) the narrow-slit configuration with small gauge and (*b*) wide-slit configurations (AD – angular dispersion; ED – energy dispersive) for which the information depth 〈*z*〉^Lap^ is defined by equation (3) and *D* denotes the thickness of the sample.

**Figure 2:**
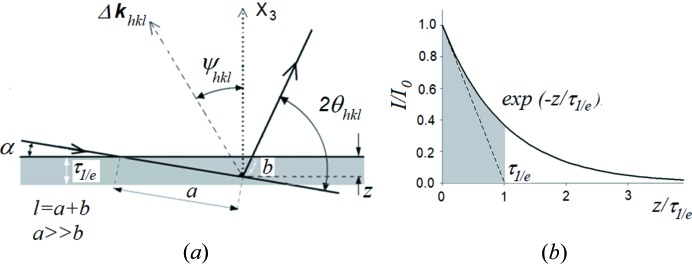
Geometry of the multireflection grazing incidence diffraction method. (*a*) The incidence angle *α* is fixed during measurement while the orientation of the scattering vector is characterized by the angle ψ_*hkl*_. (*b*) Variation of the beam intensity with the depth (*z*) below the surface and definition of information depth τ_1/*e*_.

To achieve this, two different techniques can be applied. The first is based on the definition of the so-called ‘gauge volume’, defined by slit configurations in both the primary and the diffracted beam optics and used to study stress heterogeneity inside the sample by means of neutron diffraction (millimetre scale; see *e.g.* Hutchings *et al.*, 2005 ▸) or high-energy synchrotron radiation (usually a scale of tens of micrometres or even less; see *e.g.* Allen *et al.*, 1985 ▸; Reimers *et al.*, 1998 ▸; Withers & Webster, 2001 ▸; Rowles, 2011 ▸; Genzel *et al.*, 2011 ▸). For a gauge fully immersed in the sample, the information depth 〈*z*〉^R^ is defined in real space by the absorption-weighted centroid of the gauge (see *e.g.* Meixner *et al.*, 2013 ▸). In this method, narrow slits on the incident and diffracted beams define the height of the gauge volume *h*_gv_, which should be significantly smaller than the range of studied depths 〈*z*〉^R^ as shown in Fig. 1 ▸(*a*) (Genzel *et al.*, 2012 ▸; Meixner *et al.*, 2013 ▸). Another technique for analysing the stress gradient in the near-surface region is the Laplace-space method, in which the information depth is defined by the exponential attenuation of the X-ray beam. In this case, the geometric effect of the beam size can be neglected when the configurations shown in Fig. 1 ▸(*b*) are used (Genzel *et al.*, 2007 ▸, 2012 ▸; Meixner *et al.*, 2013 ▸). The angular dispersion (AD) configuration with wide initial and diffracted beams is usually applied in cases of high absorption of low-energy X-rays at the laboratory diffractometer (*e.g.* parallel beam geometry, used in this work; see Fig. 2 ▸). For the higher-energy radiation used in the energy dispersive (ED) technique, wide slits on the incident beam and narrow slits on the diffracted beam are applied. In the latter configurations [AD and ED shown in Fig. 1 ▸(*b*)], the information depth 〈*z*〉^Lap^ is much smaller than the height of the gauge volume (*h*_gv_) immersed in the sample and defined by the slits.

In the case of the Laplace-space methods used in the present work, the position at which the stresses are determined is defined by the distribution as a function of depth of the so-called ‘diffraction power’. According to Klaus & Genzel (2013 ▸), each sublayer d*z* at a depth *z* below the surface contributes
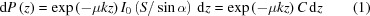
to the total diffraction signal, where 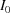 is the intensity of the incident beam, *C* = *I*_0_(*S*/sinα), μ is the linear X-ray absorption coefficient, *S* is the unit beam cross section of the incident beam, *k* is a geometry factor relating the geometrical path of the X-ray beam within the sample to the depth *z* and *α* is the angle of incidence formed by the incident beam (see also Fig. 2 ▸ *a*).

Hence, for a sample of thickness *D*, one finds
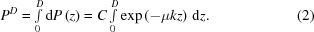
For a thick sample where 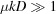, equation (2) yields 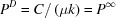.

On the basis of the above relation, an average information depth 〈*z*〉^Lap^ can be defined to which the measured X-ray signal can be assigned as the ‘centroid’ or ‘weighted average’ of the diffraction power:
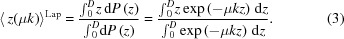
For a thick sample (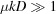) one obtains
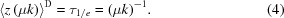
The physical interpretation of the so-called ‘information depth’ τ_1/*e*_ follows directly from equation (2) (see also Fig. 2 ▸ *b*). Accordingly, the diffraction power of a thin surface layer of thickness 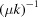 amounts to
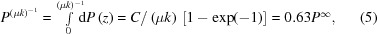
which is 63% of the diffraction power of an infinitely thick sample.

To measure the stress gradient, its effect on the measured lattice strains during sample tilt or rotation was analysed (*e.g.* Hauk, 1997 ▸; Genzel, 1999 ▸; Ruppersberg *et al.*, 1989 ▸; Klaus *et al.*, 2009 ▸; Klaus & Genzel, 2013 ▸). Alternatively, the measurements were performed for sample orientations for which the information depth was kept constant (Kumar *et al.*, 2006 ▸; Erbacher *et al.*, 2008 ▸; de Buyser *et al.*, 1991 ▸; Van Acker *et al.*, 1994 ▸; Quaeyhaegens *et al.*, 1995 ▸; Skrzypek *et al.*, 2001 ▸; Marciszko *et al.*, 2017 ▸). To characterize stress variation, different information depths were chosen by setting appropriate conditions for the experiment (usually the incident angle or energy of the X-rays). The method of combining the geometric effects of gauge volume and the effect of beam attenuation was also proposed by Meixner *et al.* (2013 ▸).

The choice of the methods presented in this work was driven by the idea of presenting a non-destructive experimental tool that allows the analysis of the residual stress gradient in the near-surface volume and the evaluation of the depth dependence of the *a*_0_ lattice parameter, as considered by Klaus & Genzel (2017 ▸) for cubic materials. In the present work, the multireflection and multiwavelength X-ray diffraction (MMXD) method of stress determination based on the Laplace-space technique will be introduced. This method allows for non-destructive analysis of residual stresses, the strain-free *a*_0_ parameter and the *c*_0_/*a*_0_ ratio (for hexagonal materials) as a function of the depth penetrated by X-rays. The results obtained from MMXD will be compared with results from the multireflection grazing incidence X-ray diffraction method (MGIXD; see *e.g.* Skrzypek *et al.*, 2001 ▸; Marciszko *et al.*, 2017 ▸), which uses only one wavelength (but multiple *hkl* reflections). A comparison will also be made with results of the ED (see *e.g.* Genzel *et al.*, 2007 ▸) diffraction technique using one *hkl* reflection and multiple wavelengths and energies, enabling us to alter the information depth.

Each of the three aforementioned Laplace-space methods exhibits advantages and disadvantages. MGIXD is the simplest method used, especially on laboratory diffractometers for low-energy X-rays (De Buyser *et al.*, 1991 ▸; Van Acker *et al.*, 1994 ▸; Quaeyhaegens *et al.*, 1995 ▸; Skrzypek *et al.*, 2001 ▸; Skrzypek & Baczmanski, 2001 ▸; Marciszko *et al.*, 2013 ▸). An important advantage of MGIXD is that it allows determination of not only the stresses but also the *a*_0_ strain-free lattice parameter and the *c*_0_/*a*_0_ ratio (Marciszko *et al.*, 2016 ▸). When higher energies are used, the number of available *hkl* reflections is not sufficient to determine stresses, and thus the range of available information depth is limited. On the other hand, the ED (standard, one reflection) method can be used to determine stresses for much greater depths; however, the information depth varies during measurement (Genzel *et al.*, 2007 ▸). The purpose of MMXD is to combine the advantages of the MGIXD and ED techniques in order to study information depth, which is constant during stress determination, and simultaneously to increase the range of depths for which the stresses can be measured. Moreover, the combination of the two methods will allow us to determine the variation of both the *a*_0_ parameter and *c*_0_/*a*_0_ ratio with depth. These goals can be achieved in MMXD, as demonstrated in this work. However, the new method is more complex than the classical ED and MGIXD techniques, which in turn leads to greater uncertainty in the determined stresses. The comparison of the three methods will be made on the basis of experimental results obtained using a laboratory diffractometer as well as synchrotron radiation to measure the stress gradients in mechanically treated Ti samples.

### 1.1. Multireflection grazing incidence diffraction method   

The MGIXD method (De Buyser *et al.*, 1991 ▸; Van Acker *et al.*, 1994 ▸; Skrzypek *et al.*, 2001 ▸; Baczmański *et al.*, 2004 ▸; Marciszko *et al.*, 2013 ▸) is an indispensable tool for non-destructive analysis of heterogeneous stresses for different (well defined) volumes below the surface of a sample. Such measurements are possible because of the small and constant angle between the incident beam (*α*) and the sample surface (see Fig. 2 ▸ *a*). Consequently, the information comes from a constant penetration depth of X-ray radiation in the studied material. The information depth can be changed by setting different incident angles. In the case of the MGIXD method, the lattice strains are measured in different crystallographic directions and are then used in the X-ray stress analysis. As mentioned above, the gradient of residual stresses in surface layers (De Buyser *et al.*, 1991 ▸; Predecki *et al.*, 1993 ▸; Van Acker *et al.*, 1994 ▸; Skrzypek *et al.*, 2001 ▸; Welzel *et al.*, 2005 ▸; Kumar *et al.*, 2006 ▸; Genzel, 1994 ▸; Genzel *et al.*, 1999 ▸; Skrzypek & Baczmanski, 2001 ▸) as well as strain-free *a*_0_ (Baczmański *et al.*, 2004 ▸) and *c*_0_/*a*_0_ parameters (for hexagonal crystals) and their depth-dependent variation can be determined (Marciszko *et al.*, 2016 ▸).

In MGIXD, the wide range of scattering vector inclinations enables us to obtain an 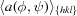 *versus* sin^2^ψ plot (where 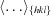 signifies the average over a series of symmetrically equivalent planes {*hkl*}) that can be used to calculate the stress tensor from linear regression or by the least-squares method (Noyan & Cohen, 1987 ▸). To perform stress measurements for a constant information depth (τ_1/*e*_) in the MGIXD method, the orientation of the scattering vector, characterized by angle *ψ*, is varied and the small angle *α* is kept constant (see Fig. 2 ▸ *a*) (De Buyser *et al.*, 1991 ▸; Van Acker *et al.*, 1994 ▸; Quaeyhaegens *et al.*, 1995 ▸; Skrzypek *et al.*, 2001 ▸; Skrzypek & Baczmanski, 2001 ▸; Marciszko *et al.*, 2013 ▸). The different orientations of the scattering vector are given by the equation
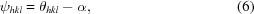
where 2*θ_hkl_* are the diffraction angles corresponding to the *hkl* reflections from which the diffraction peaks are measured.

Stresses are determined using the fundamental equations of XSA (Noyan & Cohen, 1987 ▸; Hauk, 1997 ▸; Reimers *et al.*, 2008 ▸; Welzel *et al.*, 2005 ▸) from the interplanar spacings 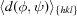 measured in the direction of the scattering vector, *i.e.* in this case, for different *ψ_hkl_* (and consequently various *θ_hkl_* angles) and for constant *α* (Fig. 2 ▸ *a*). However, in the case of the multireflection method, instead of 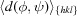, the equivalent lattice parameters 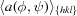 are expressed in terms of the macro­stresses *σ_ij_* and strain-free lattice constant *a*_0_ (Skrzypek *et al.*, 2001 ▸):
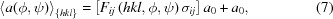
where
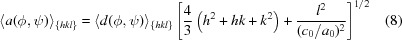
for a hexagonal structure.

*F_ij_*(*hkl*, ϕ, ψ) are the X-ray stress factors (XSFs are defined and used by Dölle & Hauk, 1978 ▸; Brakman, 1983 ▸; Barral *et al.*, 1983 ▸; Ortner, 2006 ▸). *ϕ* and *ψ* are the azimuthal and polar angles defining the orientation of the scattering vector [the possible values of *ψ* depend on the diffraction angles 2*θ_hkl_* corresponding to the available reflections *hkl*; see equation (6)].

In equations (7) and (8) the contribution of second-order plastic incompatibility stresses caused by anisotropy of the plastic deformation (for details see *e.g.* Greenough, 1949 ▸; Hauk *et al.*, 1988 ▸; Baczmański *et al.*, 1994 ▸, 2003 ▸, 2008 ▸; Gloaguen *et al.*, 2013 ▸) was neglected. These stresses can significantly influence the results of lattice strain measurements in the case of monotonic plastic deformation like the cold rolling process or tensile test. However, in the case of mechanical polishing or grinding, the plastic incompatibility stresses exhibit an approximately random orientation distribution, and, as shown by Marciszko *et al.* (2016 ▸), they increase the uncertainty of the results but do not significantly change the values of the stresses and stress-free lattice parameters determined using the MGIXD method.

In the case of hexagonal structure the value of the *c*_0_/*a*_0_ parameter is, in principle, unknown and there are two ways of calculating 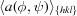 from the measured 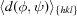. In the first, the *c*_0_/*a*_0_ ratio measured in another experiment or taken from the literature is introduced into equation (8). In the second, the iteration procedure proposed by Marciszko *et al.* (2016 ▸) for *c*_0_/*a*_0_ determination can be used. In the first step of this procedure, we substitute a theoretical value of *c*_0_/*a*_0_ into equation (8) and the least-squares method is used to find *σ_ij_* and *a*_0_ from equation (7). The result of the first adjustment is usually poor because the experimental 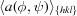 are not correctly calculated with the assumed value of *c*_0_/*a*_0_. Consequently, the experimental 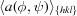 disagree with those obtained from equation (7) for optimized σ_*ij*_ and *a*_0_ fitting parameters. Hence, the procedure must be developed in order to correct the value of *c*_0_/*a*_0_ for the studied material, taking into account the macrostresses present in the sample. In this context, equation (8) can be rewritten in the following form:
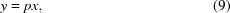
where 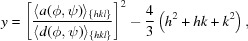


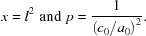



The above linear equation *versus* *l* ^2^ allows us to determine *p* and consequently *c*_0_/*a*_0_ using a simple linear regression method. The measured 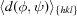 spacings and values of 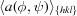 calculated from equation (7) (for *σ_ij_* and *a*_0_ optimized in the first step for an approximate value of *c*_0_/*a*_0_) are substituted. The obtained *c*_0_/*a*_0_ parameter is still an approximation, but can be applied in the second step of iteration to calculate 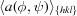 used in the least-squares procedure based on equation (8). As a result, new values of *σ_ij_* and *a*_0_ are determined. Two iteratively applied fitting procedures usually lead to convergence, allowing determination of macrostresses *σ_ij_*, the strain-free lattice parameter *a*_0_ and a more accurate value of *c*_0_/*a*_0_. Finally, if the iterative calculations converge, a very good agreement between the estimated values of 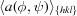 [obtained from equation (7)] and the experimental values [determined from equation (8)] can be achieved. However, note that the aforementioned procedure can be applied only when a sufficient number of experimental *hkl* are available. If this condition is not fulfilled, the known *c*_0_/*a*_0_ value must be introduced into equation (8).

As mentioned above, in the case of monochromatic X-ray radiation, the information depth (τ_1/*e*_) can be changed by an appropriate setting of the *α* angle in order to investigate materials to different depths below the sample surface (of the order of a few micrometres or even less than 1 µm). The information depth is directly determined by the attenuation of the radiation in the studied material and it is not limited by the apertures of the incident and diffracted beams [see AD configuration in Fig. 1 ▸(*b*)]. In this case, the information depth (τ_1/*e*_) can be expressed as
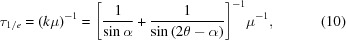
where *k* is the geometry factor and *μ* is the linear X-ray absorption coefficient which is dependent on the energy of X-rays used [equation (1)].

The relative attenuation of the scattered beam intensity (*I*/*I*_0_) as a function of the depth below the surface (*z*/τ_1/*e*_, *i.e.* related to the given value of τ_1/*e*_) is shown in Fig. 2 ▸(*b*). When different wavelengths of X-rays are used in the experiment, the information depth is defined by setting an appropriate *α* angle for the given X-ray energy (see Marciszko *et al.*, 2017 ▸).

Using equation (7) and assuming relaxation of the stress perpendicular to the surface, σ_33_(τ_1/*e*_) = 0, the other components of the stress tensor as well as the *c*_0_/*a*_0_ and *a*_0_ parameters can be determined for a given information depth τ_1/*e*_. To do this, a least-squares fitting based on equations (7) and (9) is used (Marciszko *et al.*, 2016 ▸).

In the present study, the MGIXD method was applied to measure depth-dependent profiles of stresses, *a*_0_ and *c*_0_/*a*_0_ for hexagonal crystal structures. The XSFs were calculated from single-crystal elastic constants and crystallographic texture by adopting the Eshelby–Kröner grain-interaction model (Eshelby, 1957 ▸; Kröner, 1961 ▸; Sprauel & Barral, 1989 ▸; Baczmanski *et al.*, 2003 ▸, 2008 ▸).

### 1.2. Energy dispersive synchrotron diffraction method   

ED synchrotron measurements provide complete diffraction spectra for a fixed detector position. Bragg reflections are obtained for different X-ray energies (wavelengths) so each reflection corresponds to a different depth in the sample [the experimental setup is described by Genzel *et al.* (2007 ▸)]. The residual stress analysis is based on the measurement of diffraction line profiles and the evaluation of strains for different orientations of the scattering vector. In the case of ED with a white synchrotron beam, measurements are performed for fixed 2θ diffraction angles, and the interplanar spacings 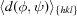 corresponding to each energy *E*(*hkl*) of the diffraction lines can be expressed as (Genzel *et al.*, 2007 ▸)
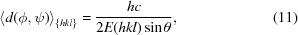
where *h* is Planck’s constant and *c* is the speed of light.

Therefore the lattice strain 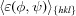 is given by
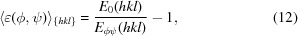
where *E*_0_(*hkl*) is the energy corresponding to the strain-free lattice spacing *d*_0_ for {*hkl*} planes and the lattice strain is determined for a given orientation (ϕ, ψ) with respect to the sample system.

In the same way as for the MGIXD method, the relation between the elastic lattice strain, measured from the diffraction spectra, and the averaged residual stress is given by the fundamental theory and equations of XSA, which were described in detail in previous work (*e.g.* Noyan & Cohen, 1987 ▸; Hauk, 1997 ▸; Reimers *et al.*, 2008 ▸).

Each *E*(*hkl*) of a reflection on the energy scale corresponds to a different (average) information depth; the symmetrical *ψ* mode (sample tilt axis in the diffraction plane) used in this work can be calculated as
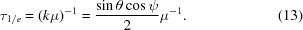
In the presented ED technique (as in the MGIXD method), the information depth is limited by the attenuation of radiation (see Figs. 1*b* and 2*b*) and it can be defined by the angles 2θ, ψ and the energy-dependent coefficient μ(*E*) (the gauge volume is not determined by the size of the incident or diffracted beam). This method of residual stress determination using ED synchrotron diffraction has been widely described in the literature (*i.e.* Genzel *et al.*, 2007 ▸) and applied to solve various special and complex problems.

### 1.3. Multireflection and multiwavelength X-ray diffraction method   

The idea of the proposed MMXD method in residual stress analysis is based on an ED diffraction measurement combined with a sin^2^ψ analysis for multiple *hkl* reflections (like in the MGIXD method). An appropriate data treatment based on the application of ED synchrotron X-ray diffraction was introduced for the analysis of stress gradients. In this case, data are collected for a constant 2θ angle like in classical stress analysis, but with a white synchrotron beam. Then the data are grouped for strictly chosen information depths. By these means, it is possible to perform the residual stress analysis layer by layer in the sample and get a much deeper profile than in the classical MGIXD method. The benefit of this approach, in contrast with classical ED stress analysis, is that the depth profile is not averaged over a wide range of depth, and multiple *hkl* reflections are used (multireflection). The constant information depth for a selected group of measured points is still expressed by equation (13), and the stresses as well as the *c*_0_/*a*_0_ and *a*_0_ parameters are calculated as in the MGIXD method, according to equations (7) and (9).

## 2. Experimental   

The preliminary experiments were performed for mechanically treated samples of Ti (grade 2, composition given in Table 1 ▸) using a Philips X-Pert X-ray diffractometer (Cu *K*α radiation) equipped with a Göbel mirror in the incident beam optic. These samples were then investigated by applying the ED synchrotron diffraction method at BESSY (EDDI@BESSYII beamline) (Genzel *et al.*, 2007 ▸; Klaus & Garcia-Moreno, 2016 ▸).

### 2.1. Sample preparation and characterization   

For testing the XSA methods, a hexagonal material with low crystal elastic anisotropy was chosen. These conditions were fulfilled in the case of Ti (grade 2), for which single-crystal elastic constants are given in Table 2 ▸. Mechanical surface treatments were selected to introduce residual stress of the opposite sign (*i.e.* compressive and tensile residual stresses), with the aim of confirming the method’s applicability in both cases. Therefore, the first Ti sample was ground and the second was mechanically polished. Grinding was supposed to introduce tensile stresses into the near-surface layers whereas polishing should introduce compressive stresses.

In the case of grinding, the rotational speed of the grinding wheel (external diameter of 300 mm, internal diameter of 127 mm and width of 40 mm) was 2000 r min^−1^ and the work speed was 9 m min^−1^. Several passes were carried out and in each pass a layer of 20 µm was removed. Manual bi-directional polishing was performed for the second sample in five steps using the following emery papers: 800, 1200, 2000, 2500, 4000 grit. The last treatment was performed with a pressing force of 5 N. Polishing paste was used for the final treatment (size of the polished surface: 1.5 × 1.5 mm). The surface roughness (*R*_a_) parameter for all mechanically treated samples is given in Table 3 ▸.

**Figure 3:**
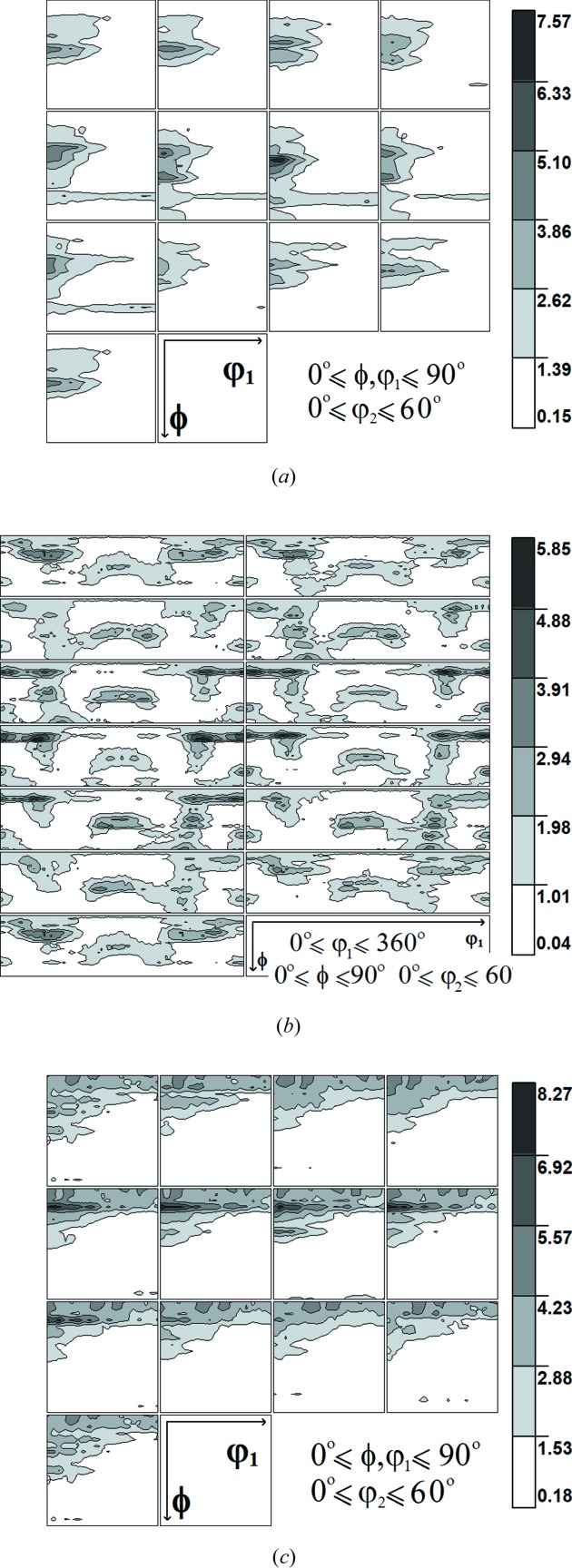
Orientation distribution function determined using Cu radiation for (*a*) the initial Ti, (*b*) ground Ti and (*c*) polished Ti samples. The sections through Euler space (Bunge, 1982 ▸) with a step of 5° are presented along the *φ*_2_ axis.

For the investigated samples (ground, polished and initial Ti) the {002}, {100}, {101} and {102} pole figures were measured on a Philips X-Pert X-ray diffractometer using Cu *K*α radiation. From the set of all measured pole figures the orientation distribution functions (ODFs; Bunge, 1982 ▸) representing crystallographic textures were determined for each sample using the WIMV method (Kallend *et al.*, 1990 ▸). As seen in Fig. 3 ▸, the grinding process changes the texture significantly and leads to lower sample symmetry (triclinic) in comparison with the initial sample (orthorhombic). Polishing also modifies the texture but these changes are smaller, *i.e.* the preferred texture orientations in Ti (grade 2) are shifted with respect to the initial orientations after polishing. Polished Ti exhibits orthorhombic sample symmetry, like the initial sample. Therefore, in these cases, the ODFs within the range 0 ≤ φ_1_ ≤ 90° are shown.

### 2.2. Laboratory classical monochromatic diffractometer   

MGIXD measurements were initially performed on a Philips X-Pert X-ray diffractometer using Cu *K*α radiation. The data were collected in continuous scan mode, integrating counts for a step size of 0.02° with a time of 6 s per step. The parallel beam configuration was used in the measurements. The incident beam optics were equipped with a Göbel mirror and Soller slit (0.04 rad) with a fixed divergence slit (0.5°), whereas the diffracted beam optics were equipped with a parallel plate collimator (0.18°) and Soller slit (0.04 rad). This configuration made it possible to use a linear focus of the X-ray tube. The advantage of the parallel beam configuration is the high resolution in determination of peak position and minimization of the error caused by sample displacement in the **z** direction. Diffraction spectra were collected for ϕ = 0 and 90° using a proportional point detector. The range of *ψ* angle was 0–70° and the 2θ scanning range was 30–150°. Measurements were performed for two incidence angles *α* = 5 and 15°. To exclude geometrical errors in peak shifts resulting from diffractometer misalignment, the powder reference sample was also measured under the same conditions as the examined samples. The shapes of the diffraction peaks were corrected for the Lorentz–polarization factor as well as for absorption effects (LPA correction) using appropriate formulas for MGIXD, given by Wroński *et al.* (2009 ▸) and Marciszko (2013 ▸). Pseudo-Voigt profiles (taking into account the *K*α_1_ + *K*α_2_ doublet) with a linear background approximation were fitted to well defined and good quality peaks in order to determine their 2θ positions. We found that for the analysed diffraction patterns the LPA correction does not significantly change the peak positions and the results of stress measurements.

### 2.3. Synchrotron EDDI measurements   

In the next step, the multireflection method was applied for the energy dispersive method, using a white beam (wavelength λ in the range 0.89–0.31 Å, corresponding to an energy range of 13.9–40 keV). The measurements were performed on the EDDI@BESSYII beamline at the BESSY synchrotron (Berlin, Germany) in reflection geometry (Klaus & Garcia-Moreno, 2016 ▸). The synchrotron white beam was generated by the 7T-Wiggler and passed about 30 m through optical components up to the location of the sample. An absorber mask limits the beam diameter to 3.9 ×3.9 mm. A low-energy solid-state Ge detector was used to collect the diffraction data. In order to achieve the required characteristics of the beam, a system of slits and filters is provided. The primary beam cross section was defined as 0.5 × 0.5 mm. The angular divergence in the diffracted beam was restricted by a double slit system with apertures of 0.03 × 5 mm to *Δθ* ≤ 0.005°. Note that the aperture of the primary beam slit (0.5 mm) is much larger than that of the secondary beam slit system (0.03 mm). Since only half of the volume element is immersed in the material, the effective height of the gauge volume 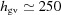
 µm, which is much larger than the information depth τ_1/*e*_ < 20 µm (see Fig. 1 ▸ *b*). Hence, the depth from which the information in the diffracted signal originates is limited by absorption and not the size of the gauge volume.

The scattering angles 2θ chosen were equal to 7, 10, 16 and 20°. Diffraction spectra were collected in symmetrical *ψ* mode for *ϕ* = 0, 90, 180 and 270°. Residual stresses were evaluated by means of the sin^2^ψ method in steps of *Δψ* = 4° (for *ψ* = 0, 72°) and *Δψ* = 2° (for *ψ* = 74, 80°). The diffraction peaks were fitted using the pseudo-Voigt function. A reference Au powder was used to exclude geometrical errors caused by apparatus misalignment.

## 3. Results and discussion   

In this work, the methodology for experimental data interpretation has been developed in order to treat data obtained not only for different incident angles but also using simultaneously different wavelengths. Therefore, the new method is not only ‘multireflection’ but also ‘multiwavelength’, and more experimental data are available to calculate the values of stresses in comparison with MGIXD. Moreover, the application of high-energy synchrotron radiation enables measurements of much deeper volumes compared with classical diffraction performed using Cu *K*α X-rays.

### 3.1. Residual stress profile – MGIXD classical X-ray measurements   

**Figure 4:**
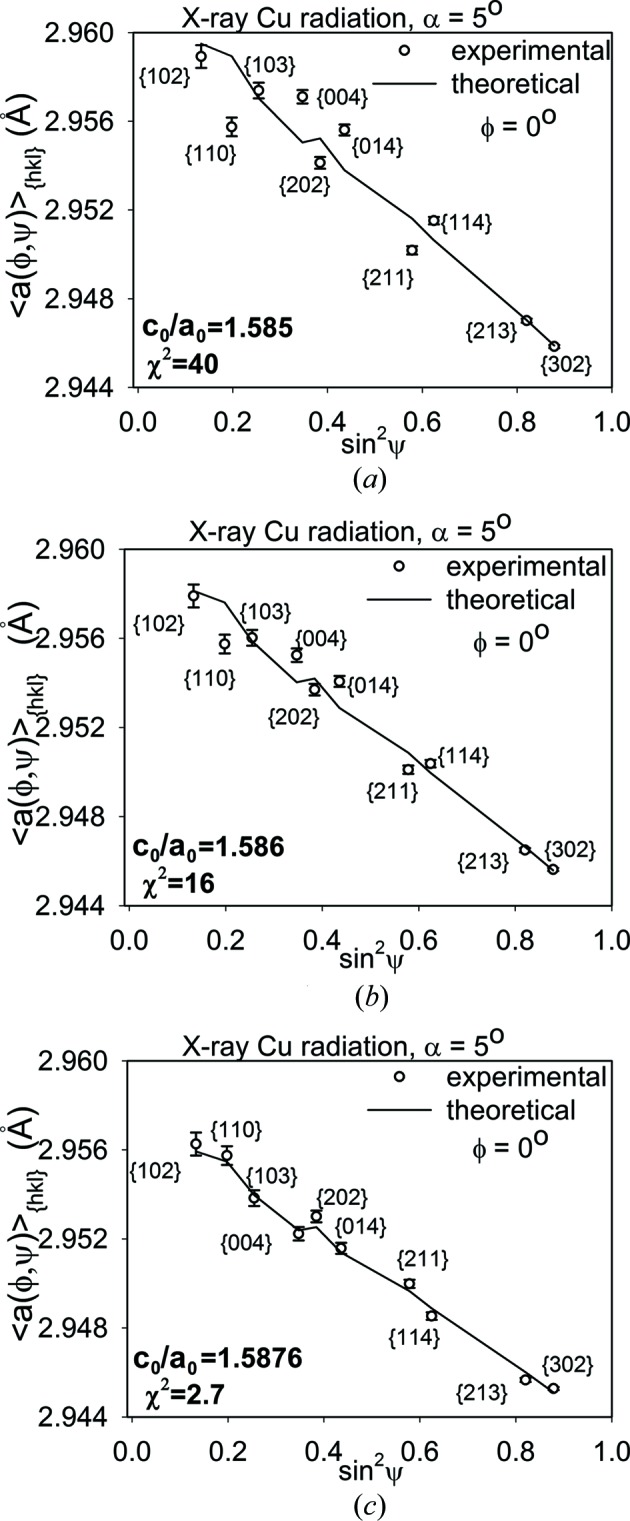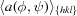 *versus* sin^2^ψ plots for the mechanically polished Ti sample, measured with α = 5°. In figures (*a*) and (*b*) the theoretical plots were fitted to the experimental points determined with assumed *c*_0_/*a*_0_ values, whereas in the case of figure (*c*) the *c*_0_/*a*_0_ parameter was adjusted. An uncertainty of the peak position δ(2θ) = 0.01° was assumed.

Firstly, the calculation of the stresses in polished and ground Ti (grade 2) was performed using the assumed values of the *c*_0_/*a*_0_ parameter indicated in Figs. 4(*a*), 4(*b*) ▸, 5(*a*) and 5(*b*) ▸. In this case, the value of *c*_0_/*a*_0_ was not varied during data treatment. Note that the experimental points are spread far from the lines obtained by fitting equation (7) with the XSFs calculated using the Eshelby–Kröner model [see 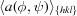 *versus* sin^2^ψ plots in Figs. 4(*a*), 4(*b*) ▸, 5(*a*) and 5(*b*)] from the single-crystal elasticity constants given in Table 2 ▸ and the ODFs shown in Fig. 3 ▸.

Next, the iterative procedure was used and the *c*_0_/*a*_0_ value was also adjusted. The resulting 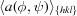 *versus* sin^2^ψ plots exhibit significantly better agreement between theoretical and experimental points (Figs. 4 ▸ *c* and 5 ▸ *c*). The values of the *c*_0_/*a*_0_ parameter and goodness of fit χ^2^ (Marciszko *et al.*, 2016 ▸) determined using the procedure presented in §1 are also given in these figures. It can be seen that the value of χ^2^ decreases significantly when the experimental points approach the theoretical curves.

**Figure 6:**
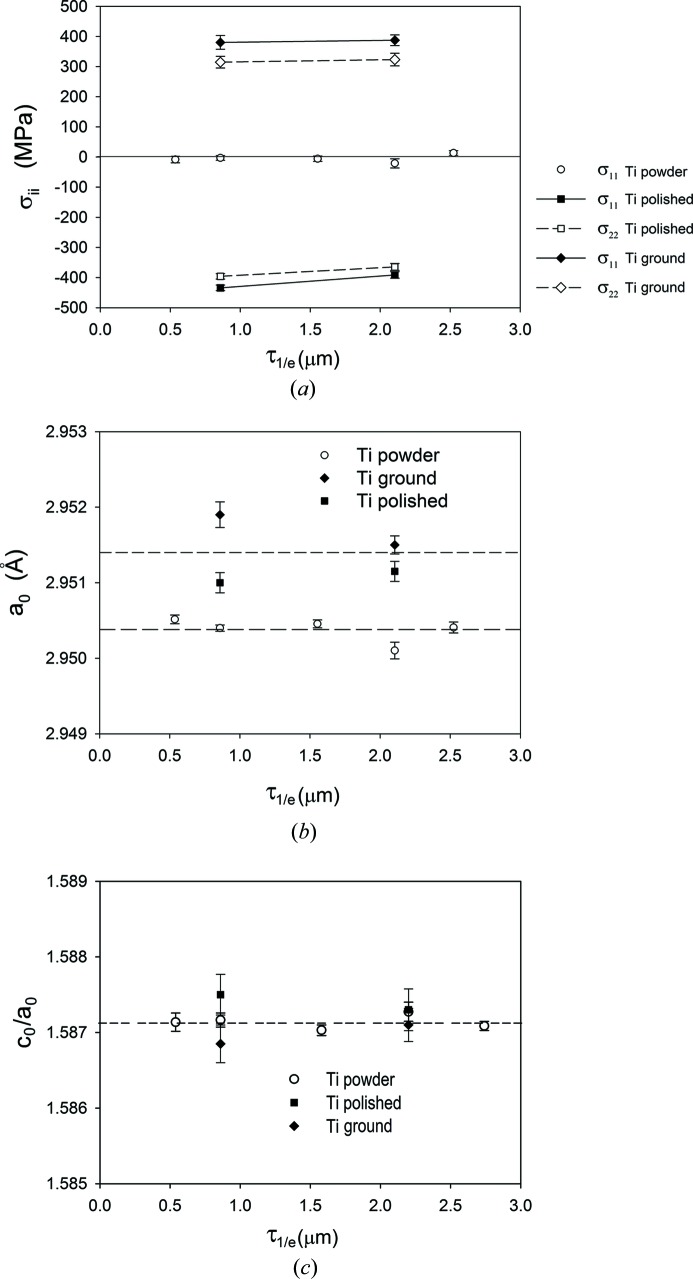
The depth-dependent profiles of stresses (*a*), *a*_0_ (*b*) and *c*_0_/*a*_0_ (*c*) for mechanically polished (bi-directional polishing) and ground (where σ_11_ is parallel to the grinding direction) Ti grade 2 samples, as well as the reference powder sample, obtained by the MGIXD method. Cu *K*α radiation and the pseudo-Voigt profile were used for fitting.

For the mechanically treated samples, the values of stresses and the *a*_0_ and *c*_0_/*a*_0_ lattice parameters were determined in the near-surface region for two information depths (τ_1/*e*_) corresponding to different incident angles (*α* = 5 and 15°), and compared with analogous measurements performed for the Ti powder sample (in the latter case more depths were studied). The results presented in Fig. 6 ▸ show that stresses close to zero were measured in the Ti powder, which means that our experimental setup and method for stress calculation are validated. Different types of stresses were generated from the two surface treatments, *i.e.* tensile stresses after grinding (slightly higher stress along the direction of grinding) and compressive stress after polishing. No significant stress evolution was observed in the depth penetrated by X-rays for ground and polished samples in the range of information depth that was accessible on the classical diffractometer. Also, no significant evolution with depth was found for *a*_0_ and *c*_0_/*a*_0_ parameters for all measured samples. The averages of the parameters calculated for both mechanically treated Ti (grade 2) samples and both incidence angles [*a*_0_ = 2.9515 (10) Å and *c*_0_/*a*_0_ = 1.5871 (4)] are close to those determined for the powder sample [*a*_0_ = 2.9503 (3) Å and *c*_0_/*a*_0_ = 1.5871 (1)], as well as to the accurate values given by Wood (1962 ▸) for high-purity Ti [*a*_0_ = 2.95111 (6) Å, *c*_0_ = 4.68433 (10) Å and *c*_0_/*a*_0_ = 1.5873] [similar values were reported by Lutjering & Williams (2003 ▸)]. Small discrepancies between *a*_0_ values measured for different samples can be caused by different levels of impurity elements in the studied Ti materials.

### 3.2. Residual stress profile – ED measurement using synchrotron radiation and new analysis   

As in the case of classical MGIXD X-ray measurements, in the synchrotron data analysis, the XSFs were calculated with the Eshelby–Kröner model using the single-crystal elastic constants given in Table 2 ▸ and the ODFs shown in Fig. 3 ▸. All of the collected diffraction peak shapes were fitted using the pseudo-Voigt function. The stress analysis based on the synchrotron measurements was performed using two different methods.

**Figure 7:**
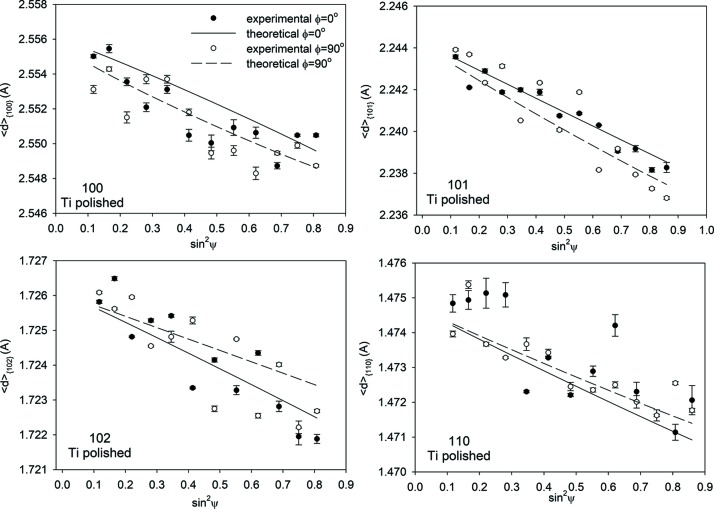
Examples of 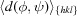 *versus* sin^2^ψ plots for the polished Ti sample obtained using standard analysis and different *hkl* reflections.

The first method of analysis was the standard sin^2^ψ method (ψ geometry) in which a constant 2θ of 16° was used. Each 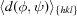 *versus* sin^2^ψ plot was measured for different *hkl* reflections. Because the absorption varies for different energies (and wavelengths) of radiation, each plot was determined for a different average information depth. However, the information depth is not constant and varies *versus* sin^2^ψ. Example 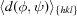 *versus* sin^2^ψ plots for standard ED analysis are shown in Figs. 7 ▸ and 8 ▸ for *ϕ* = 0 and 90° only (the determined σ_13_ and σ_23_ shear stresses are negligible).

**Figure 9:**
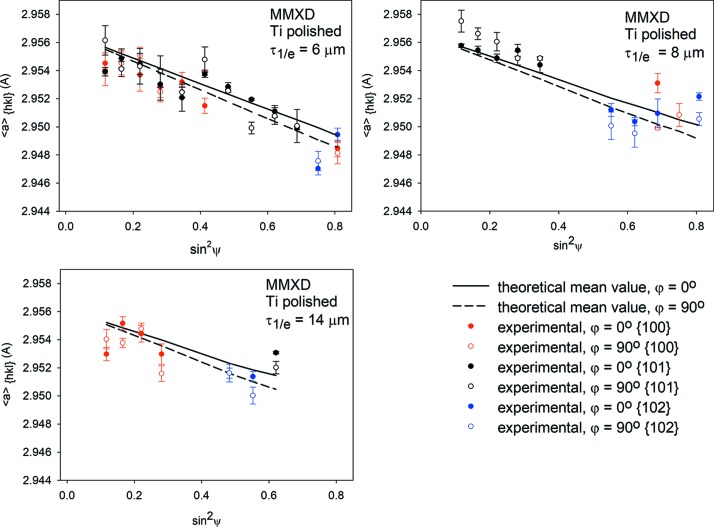
Example of 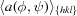 *versus* sin^2^ψ plots obtained using multireflection analysis for a polished Ti sample. The lines represent mean values of theoretical 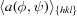 parameters over all available reflections, while the experimental data are shown using different colours for different *hkl* reflections.

**Figure 10:**
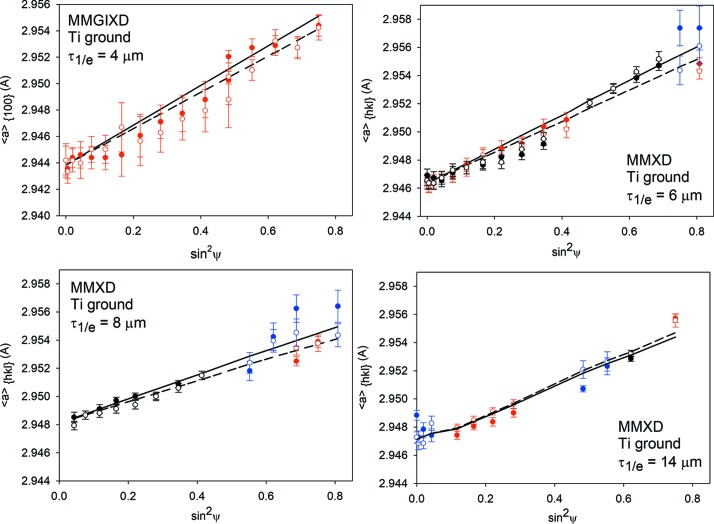
Similar results to those in Fig. 9 ▸, but for a ground Ti sample (the same legend and convention of data presentation apply).

The second method of analysis (MMXD) was based on the data obtained for four 2θ values: 7, 10, 16 and 20° for which different reflections were used. They were grouped in sets corresponding to chosen ranges of information depth with intervals of ±2 µm and the data exhibiting a large uncertainty of the determined peak position were removed. In this method, only the values of 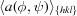 corresponding to the defined interval of information depth (different wavelengths and *hkl* reflections) were chosen to create one sin^2^ψ plot. The obtained sin^2^ψ plots are presented in Figs. 9 ▸ and 10 ▸ for different depths τ_1/*e*_ (because of a low elastic anisotropy of Ti, the mean values of the theoretical lattice parameters 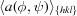 for all available reflections are shown). Note that for the information depth τ_1/*e*_ = 4 µm in the ground sample only the 100 reflection (measured for different 2θ and *ψ* angles) was used to construct the sin^2^ψ plots (Fig. 10 ▸). In this case, the interplanar parameter was not dependent on the *c*_0_/*a*_0_ ratio and so the stress values and *a*_0_ parameter were determined directly from equations (7) and (8) (as in the standard method) without adjustment of the *c*_0_/*a*_0_ value [equation (9)]. Therefore, for this depth, the *c*_0_/*a*_0_ value was not found but the *a*_0_ value was determined unambiguously. Comparing the results for both samples studied (Figs. 9 ▸ and 10 ▸) it can be concluded that the quality of experimental data obtained for the ground sample is much better in comparison with the polished one. As a result of significant plastic deformation in the surface layer of the polished material, fewer peaks were available and the uncertainty of the peak positions was greater. In the case of information depth τ_1/*e*_ = 4 µm for the polished sample, the low quality of the experimental data and large uncertainties of the measured peak positions (100 reflections) prevented a stress analysis (see Fig. 9 ▸). The sin^2^ψ plots constructed for τ_1/*e*_ = 6–14 µm in both samples consist of three (or at least two) reflections, which enabled us to apply the MMXD analysis [based on equations (7)–(9) [Disp-formula fd9]] and calculate both *c*_0_/*a*_0_ and *a*_0_ parameters.

**Figure 11:**
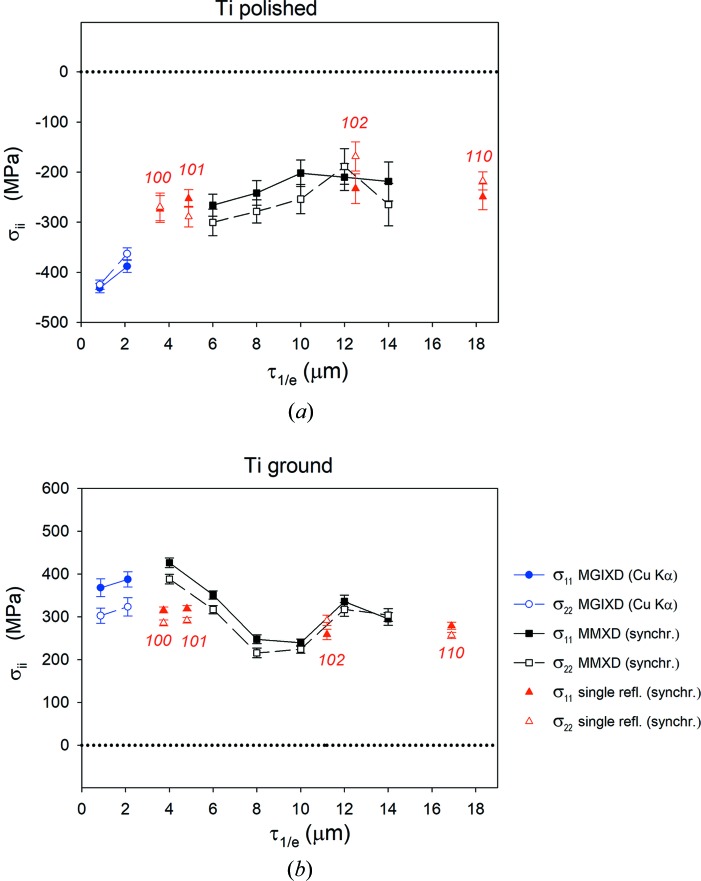
The depth-dependent profile of stresses determined for (*a*) polished and (*b*) ground samples (Ti grade 2). Comparison of the results from a classical diffractometer (MGIXD), synchrotron EDDI experiment (MMXD) and the standard ED stress measurements.

In view of the results for the stress analysis presented in Fig. 11 ▸, it can be concluded that a good convergence of the results from different methods was obtained within the range 0–18 µm of information depth. It was also found that, for the MMXD technique, the deeper the information depth, the smaller the number of reflections available for a given range of Δτ_1/*e*_ = ±2 µm and, as a consequence, the sin^2^ψ plots are constructed for a smaller range of sin^2^ψ. This defines the limit of applicability of the method. On the other hand, close to the sample surface, the stresses determined using synchrotron data agree with the results obtained on a laboratory diffractometer (using Cu *K*α radiation).

**Figure 12:**
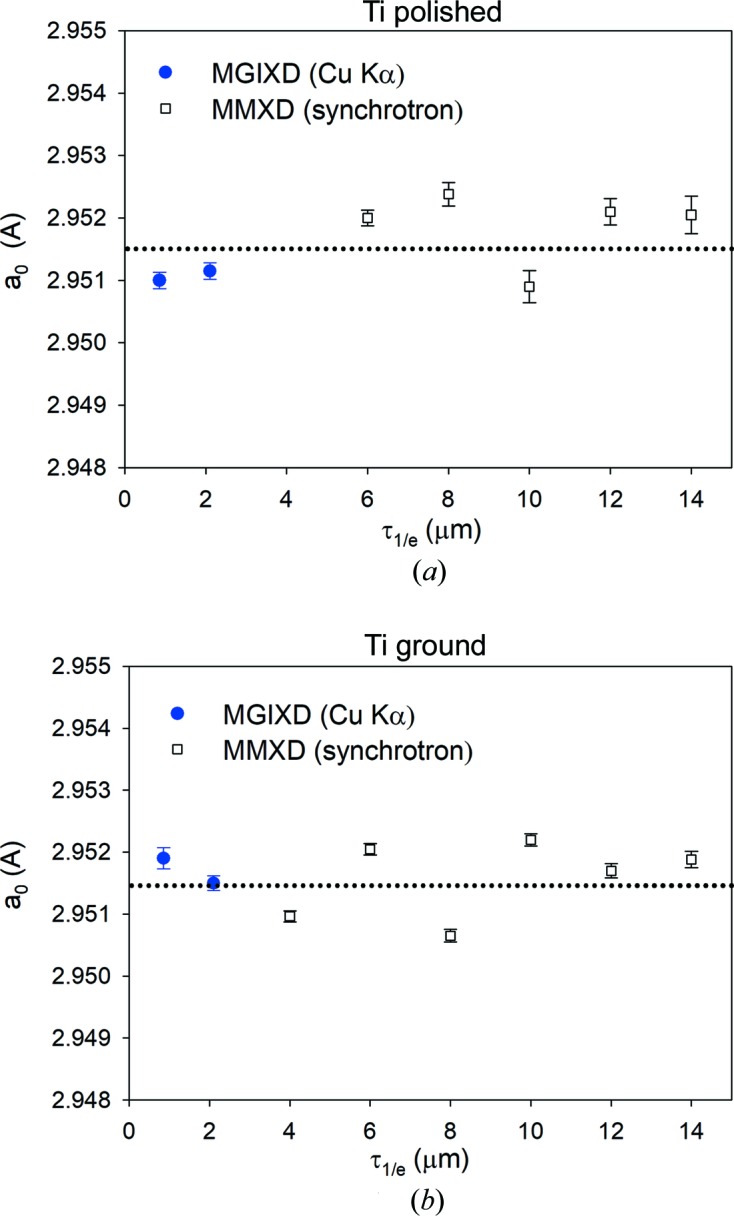
The depth-dependent profile (in the range 0–14 µm) of lattice parameter *a*_0_ for (*a*) polished and (*b*) ground samples. Comparison of the results from a classical diffractometer (MGIXD) and synchrotron EDDI experiment (MMXD).

Using the MMXD method of data analysis, it was possible to determine *a*_0_ and *c*_0_/*a*_0_ parameters for the studied range of information depth (excluding τ_1/*e*_ = 4 µm, where only *a*_0_ was determined for the ground sample). As shown in Figs. 12 ▸ and 13 ▸, both parameters are close to those obtained from MGIXD (on a laboratory diffractometer) and they do not change significantly with the information depth. The spread of the experimental results around the average values is caused by reasons such as inaccuracy of the XSFs, the limited number of reflections used and possible misalignments of the experimental setup leading to inaccurate values of the 2θ angle and *λ* wavelengths. Moreover, factors affecting the beam intensity such as diffraction extinction and crystallographic texture were not taken into account when the information depths were estimated. The issue of X-ray beam attenuation is important for the proposed methodology, because it can lead to incorrect estimation of the information depth calculated on the basis of the linear absorption coefficient *μ*. However, for ground or polished samples, the mechanical treatment significantly increases the imperfection of the crystals and minimizes extinction effects (Warren, 1969 ▸). In the samples studied here, the primary extinctions can be neglected because the size of the coherent domain determined from the Williamson–Hall method (between 20 and 50 nm; see Marciszko *et al.*, 2013 ▸) is significantly smaller than the extinction length (larger than 700 nm) calculated for the strongest reflection and for the used energy range (Zachariasen, 1945 ▸; Kryshtab *et al.*, 2004 ▸). Also, the effect of secondary extinction in diffracting grains is small because of large misorientations of the lattice within grains; a range of 1–2° was estimated from electron backscatter diffraction measurements (see Wroński *et al.*, 2015 ▸), which is larger than the limit of some arcminutes below which such effects may play a role (Zachariasen, 1963 ▸). Moreover, the overall influence of secondary extinction on the beam attenuation in polycrystalline aggregates is much lower than that calculated for a single polycrystalline grain [assuming mosaic grain structure, as in the work of Zachariasen (1963 ▸)] owing to large differences between grain orientations (even in the case of crystallographic texture). Therefore, the effect of the primary and secondary extinction as well as the texture is not significant in the case of mechanically treated surfaces but should be taken into account in deposited layers if large near-perfect crystals are present (Chaudhuri & Shah, 1991 ▸; Birkholz *et al.*, 2005 ▸).

**Figure 13:**
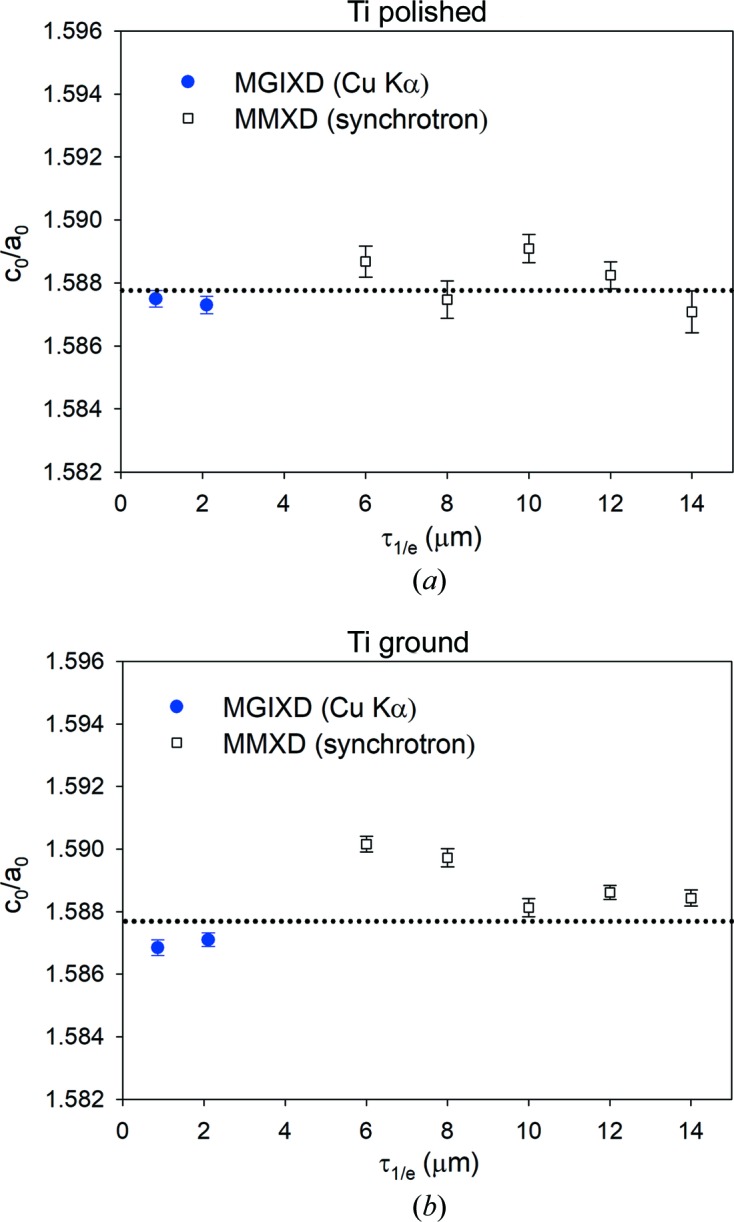
Similar comparison to that in Fig. 12 ▸, but for the determined *c*_0_/*a*_0_ ratio.

To reduce the influence of misalignment effects, calibration on the gold powder sample was performed individually for each measured peak position. We also checked that the choice of model for XSF calculations did not significantly change the results obtained. Finally, the deviation of the *a*_0_ parameter from the average value obtained with the MGIXD and MMXD methods is approximately equal to 0.001 Å (Fig. 12 ▸), whereas the *c*_0_/*a*_0_ parameter deviations are about 0.0015 (Fig. 13 ▸). In both cases, the deviations are larger than the uncertainties determined from the least-squares fitting procedure. It should be also emphasized that the values of both parameters (especially *c*_0_/*a*_0_) are more reliable in the case of the MGIXD method, in which many *hkl* reflections were used in the analysis.

From the results obtained in this study, we can clearly see the advantage of the MMXD method, in which the ED experimental data are analysed step by step for given increments of information depth. The variation of the stresses as a function of depth with steps of 2 µm within the range 4–14 µm (defined for information depth in Laplace space) can be determined and the results agree with those obtained with standard ED measurements. The standard ED method based on a single *hkl* reflection gives the average stresses integrated over a wide range of information depth (only four values of stress at different depths, measured using 2θ = 16°, are presented in Fig. 11 ▸). On the other hand, the depth-dependent stress profile is well characterized using MMXD analysis. Moreover, results obtained using MMXD with synchrotron radiation confirmed the values of stress measured close to the sample surface using MGIXD with Cu *K*α radiation, *i.e.* perfect continuity between the two ranges available for these methods was obtained. The *a*_0_ and *c*_0_/*a*_0_ lattice parameters can also be determined by applying MMXD analysis, but in order to obtain better results the quality of the diffraction data must be improved and the availability of different *hkl* reflections should be increased.

## 4. Conclusions   

In this study, a new approach for the analysis of ED synchrotron data was proposed and tested on mechanically treated Ti surfaces. This analysis (called MMXD) allowed us to determine a depth-dependent stress profile with a step of 2 µm in the Laplace space. Note that this does not mean the spatial resolution in real space is equal to this step. However, the applicability of the MMXD method for samples exhibiting a strong stress gradient is evident from the fact that the data are grouped in much smaller ranges in comparison with the standard ED method (this should be shown in future on appropriate example specimens). Special care should be taken when analysing MMXD data for samples consisting of near-perfect crystals, in which case the extinction effect should be taken into account in calculation of the information depth for which experimental points are grouped.

For mechanically treated surfaces of Ti-alloy samples, a good convergence was obtained between the stresses measured using synchrotron radiation (MMXD and standard ED methods) and those determined with Cu *K*α radiation on a laboratory diffractometer (MGIXD method). Certainly, synchrotron radiation with higher energies allowed measurements for larger depths in comparison with laboratory X-rays. The advantage of the MGIXD and MMXD methods is the possibility for determination of both *a*_0_ and *c*_0_/*a*_0_ strain-free lattice parameters in the well defined surface region of the sample.

## Figures and Tables

**Figure 5:**
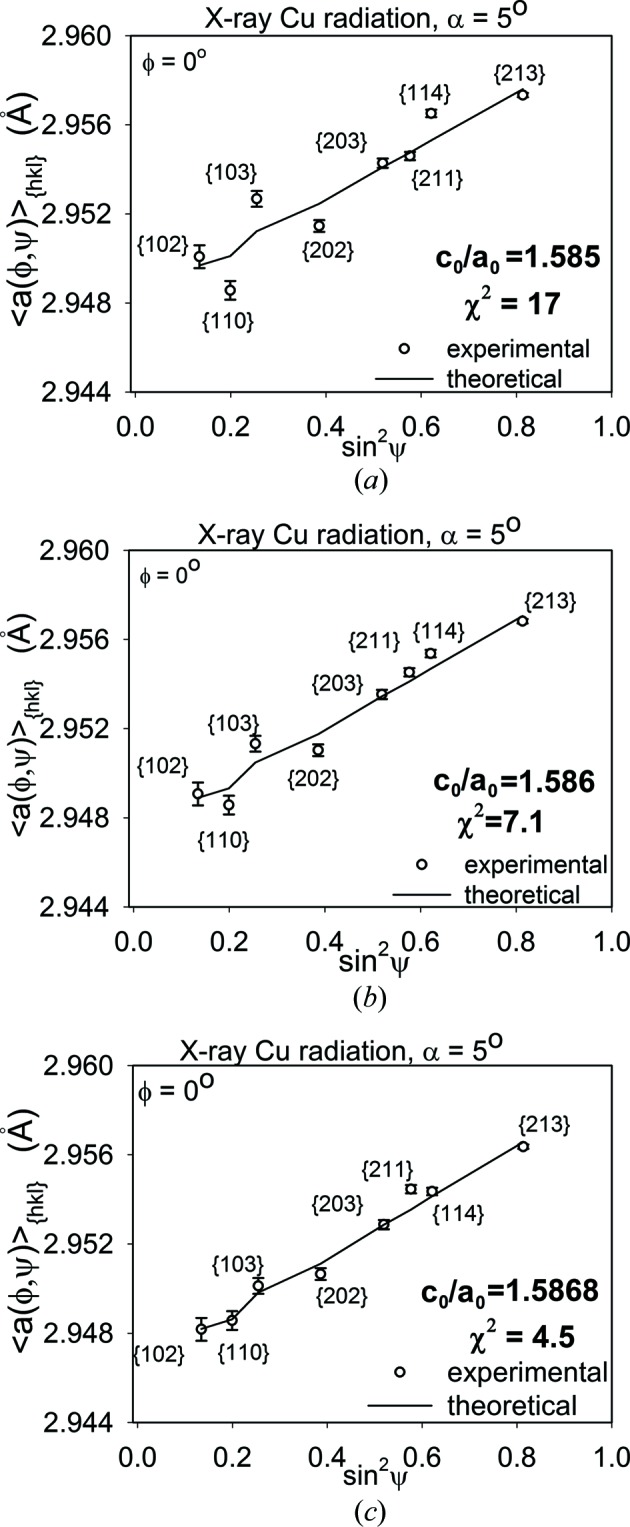
Similar results to those in Fig. 4 ▸, but for a ground Ti sample.

**Figure 8:**
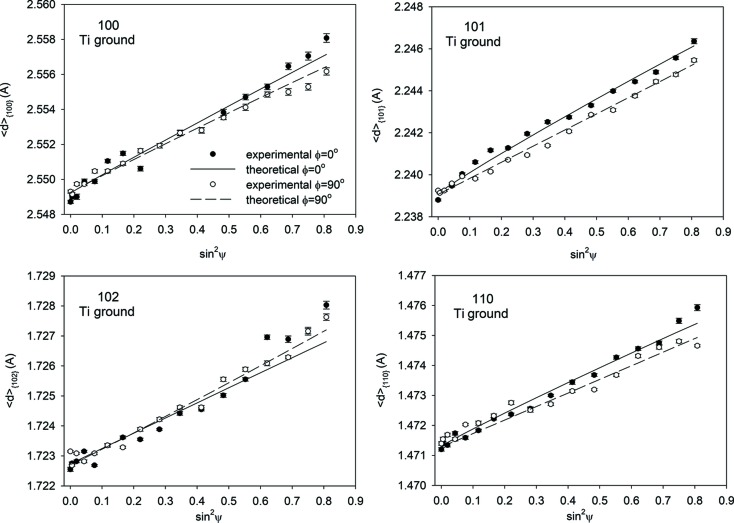
Similar results to those in Fig. 7 ▸, but for a ground Ti sample.

**Table 1:** Composition of the Ti samples (grade 2) used in the present work (wt%)

Ti	O	Fe	Ni	C	N
Balance	0.131	0.109	0.020	0.010	0.010

**Table 2:** Single-crystal elastic constants for the studied Ti (grade 2) sample (Boyer *et al.*, 1994 ▸; Simoms & Wang, 1971 ▸)

*C*_*ij*_ (GPa)	*C*_11_	*C*_12_	*C*_13_	*C*_33_	*C*_44_	*C*_66_
Ti	162	92	69	180	47	35

**Table 3:** Values of surface-roughness parameter (*R*_a_) for investigated Ti samples

Surface treatment	*R*_a_ (µm)
Polishing	0.04
Grinding	1.87

## References

[bb1] Allen, A. J., Hutchings, M. T., Windsor, C. G. & Andreani, C. (1985). *Adv. Phys.* **34**, 445–473.

[bb2] Baczmański, A., Braham, C. & Seiler, W. (2003). *Philos. Mag.* **83**, 3225–3246.

[bb3] Baczmański, A., Braham, C., Seiler, W. & Shiraki, N. (2004). *Surf. Coat. Technol.* **182**, 43–54.

[bb4] Baczmanski, A., Lipinski, P., Tidu, A., Wierzbanowski, K. & Pathiraj, B. (2008). *J. Appl. Cryst.* **41**, 854–867.

[bb5] Baczmański, A., Wierzbanowski, K., Lipiński, P., Helmholdt, R. B., Ekambaranathan, G. & Pathiraj, B. (1994). *Philos. Mag. A*, **69**, 437–449.

[bb6] Barral, M., Sprauel, J. M. & Maeder, G. (1983). *Eigenspannungen*, Vol. 1, edited by E. Macherauch & V. Hauk, pp. 31–47. Oberursel: Deutsche Gesellschaft für Metallkunde.

[bb7] Birkholz, M. (2005). *Thin Film Analysis by X-ray Scattering.* Weinheim: Wiley-VCH Verlag.

[bb8] Boyer, R., Collings, E. W. & Welsch, G. (1994). *Materials Properties Handbook: Titanium Alloys.* Materials Park: ASM International.

[bb9] Brakman, C. M. (1983). *J. Appl. Cryst.* **16**, 325–340.

[bb10] Bunge, H. J. (1982). *Texture Analysis in Materials Science: Mathematical Methods.* London: Butterworths and Co.

[bb11] Chaudhuri, J. & Shah, S. (1991). *J. Appl. Phys.* **69**, 499–501.

[bb12] De Buyser, L., Van Houtte, P. & Aernoudt, E. (1991). *Textures and Microstructures*, **14–18**, 73–78.

[bb13] Dölle, H. & Hauk, V. (1978). *Z. Metallkd.* **69**, 410–417.

[bb14] Erbacher, T., Wanner, A., Beck, T. & Vöhringer, O. (2008). *J. Appl. Cryst.* **41**, 377–385.

[bb15] Eshelby, J. D. (1957). *Proc. R. Soc. London Ser. A*, **241**, 376–396.

[bb16] Genzel, Ch. (1994). *Phys. Status Solidi (A)*, **146**, 629–637.

[bb17] Genzel, C. (1999). *J. Appl. Cryst.* **32**, 770–778.

[bb18] Genzel, C., Broda, M., Dantz, D. & Reimers, W. (1999). *J. Appl. Cryst.* **32**, 779–787.

[bb19] Genzel, Ch., Denks, I. A., Coelho, R., Thomas, D., Mainz, R., Apel, D. & Klaus, M. (2011). *J. Strain Anal. Eng. Des.* **46**, 615–625.

[bb20] Genzel, Ch., Denks, I. A., Gibmeier, J., Klaus, M. & Wagener, G. (2007). *Nucl. Instrum. Methods Phys. Res. A*, **578**, 23–33.

[bb21] Genzel, Ch., Denks, I. A. & Klaus, M. (2012). *Modern Diffraction Methods*, edited by E. J. Mittemeijer & U. Welzel, pp. 127–154. Weinheim: Wiley-VCH.

[bb22] Gloaguen, D., Oum, G., Legrand, V., Fajoui, J. & Branchu, S. (2013). *Acta Mater.* **61**, 5779–5790.

[bb23] Greenough, G. B. (1949). *Proc. R. Soc. London Ser. A*, **197**, 556–567.

[bb24] Hauk, V. (1997). *Structural and Residual Stress Analysis by Nondestructive Methods.* Amsterdam: Elsevier.

[bb25] Hauk, V., Krug, W. K., Oudelhoven, W. M. & Pintschovius, L. (1988). *Z. Metallkd.* **79**, 159–167.

[bb26] Hutchings, M. T., Withers, P. J., Holden, T. M. & Lorentzen, T. (2005). *Introduction to the Characterization of Residual Stress by Neutron Diffraction.* Boca Raton: Taylor and Francis.

[bb27] Kallend, J. S., Kocks, U. F., Rollet, A. D. & Wenk, H. R. (1990). *Operational Texture Analysis*. Report LA-UR-90-2852. Centre for Material Sciences, Los Alamos National Laboratory, New Mexico, USA.

[bb28] Klaus, M. & Garcia-Moreno, F. (2016). *J. Large-Scale Res. Facil.* **2**, A40.

[bb29] Klaus, M. & Genzel, Ch. (2013). *J. Appl. Cryst.* **46**, 1266–1276.

[bb30] Klaus, M. & Genzel, Ch. (2017). *J. Appl. Cryst.* **50**, 252–264.

[bb31] Klaus, M., Reimers, W. & Genzel, Ch. (2009). *Powder Diffr. Suppl.* **24**, 82–86.

[bb32] Kröner, E. (1961). *Acta Metall.* **9**, 155–161.

[bb33] Kryshtab, T., Palacios Gómez, J., Mazin, M. & Gómez Gasga, G. (2004). *Acta Mater.* **52**, 3027–3034.

[bb34] Kumar, A., Welzel, U. & Mittemeijer, E. J. (2006). *J. Appl. Cryst.* **39**, 633–646.

[bb35] Lutjering, G. & Williams, J. C. (2003). *Titanium Alloys*. Heidelberg: Springer.

[bb36] Marciszko, M. (2013). PhD thesis, AGH, Kraków, Poland. http://winntbg.bg.agh.edu.pl/rozprawy2/10658/full10658.pdf.

[bb37] Marciszko, M., Baczmański, A., Braham, C., Wróbel, M., Seiler, W., Wroński, S. & Berent, K. (2016). *J. Appl. Cryst.* **49**, 85–102.

[bb38] Marciszko, M., Baczmański, A., Braham, C., Wróbel, M., Wroński, S. & Cios, G. (2017). *Acta Mater.* **123**, 157–166.

[bb39] Marciszko, M., Baczmański, A., Wróbel, M., Seiler, W., Braham, C., Donges, J., Śniechowski, M. & Wierzbanowski, K. (2013). *Thin Solid Films*, **530**, 81–84.

[bb40] Meixner, M., Klaus, M. & Genzel, Ch. (2013). *J. Appl. Cryst.* **46**, 610–618.

[bb41] Noyan, I. C. & Cohen, J. B. (1987). *Residual Stress. Measurement by Diffraction and Interpretation*. New York: Springer.

[bb42] Ortner, B. (2006). *J. Appl. Cryst.* **39**, 401–409.

[bb43] Predecki, P., Ballard, B. & Zhu, X. (1993). *Adv. X-ray Anal.* **36**, 237–245.

[bb44] Quaeyhaegens, C., Knuyt, G. & Stals, L. M. (1995). *Surf. Coat. Technol.* **74–75**, 104–109.

[bb45] Reimers, W., Broda, M., Brusch, G., Dantz, D., Liss, K.-D., Pyzalla, A., Schmackers, T. & Tschentscher, T. (1998). *J. Nondestr. Eval.* **17**, 129–140.

[bb46] Reimers, W., Pyzalla, A., Schreyer, A. & Clemens, H. (2008). *Neutrons and Synchrotron Radiation in Engineering Materials Science: From Fundamentals to Material and Component Characterization.* Weinheim: Wiley-VCH.

[bb47] Rowles, M. R. (2011). *J. Synchrotron Rad.* **18**, 938–941. 10.1107/S0909049511033267 21997921

[bb48] Ruppersberg, H., Detemple, I. & Krier, J. (1989). *Phys. Status Solidi (A)*, **116**, 681–687.

[bb49] Simoms, G. & Wang, H. (1971). *Single Crystal Elastic Constants and Calculated Aggregate Properties: a Handbook*, 2nd ed. Cambridge: The MIT Press.

[bb50] Skrzypek, S. J. & Baczmanski, A. (2001). *Adv. X-ray Anal.* **44**, 124–145.

[bb51] Skrzypek, S. J., Baczmański, A., Ratuszek, W. & Kusior, E. (2001). *J. Appl. Cryst.* **34**, 427–435.

[bb52] Sprauel, J. M., Francois, M. & Barral, M. (1989). *International Conference on Residual Stresses, ICRS2*, edited by G. Beck, S. Denis & A. Simon, pp. 172–177. London, New York: Elsevier Applied Science.

[bb53] Van Acker, K., De Buyser, L., Celis, J. P. & Van Houtte, P. (1994). *J. Appl. Cryst.* **27**, 56–66.

[bb54] Warren, B. (1969). *X-ray Diffraction.* Reading: Addison–Wesley Publishing Company.

[bb55] Welzel, U., Ligot, J., Lamparter, P., Vermeulen, A. C. & Mittemeijer, E. J. (2005). *J. Appl. Cryst.* **38**, 1–29.

[bb56] Withers, P. J. & Webster, P. J. (2001). *Strain*, **37**, 19–33.

[bb57] Wood, R. M. (1962). *Proc. Phys. Soc.* **80**, 783–786.

[bb59] Wroński, M., Wierzbanowski, K., Wróbel, M., Wroński, S. & Bacroix, B. (2015). *Met. Mater. Int.* **21**, 805–814.

[bb58] Wroński, S., Wierzbanowski, K., Baczmański, A., Lodini, A., Braham, C. & Seiler, W. (2009). *Powder Diffr. Suppl.* ** 24**, S1–S15.

[bb60] Zachariasen, W. H. (1945). *Theory of X-ray Diffraction in Crystals.* New York, London: J. Wiley/Chapman and Hall.

[bb61] Zachariasen, W. H. (1963). *Acta Cryst.* **16**, 1139–1144.

